# Facile spectrophotometric assay of molar equivalents of *N*-hydroxysuccinimide esters of monomethoxyl poly-(ethylene glycol) derivatives

**DOI:** 10.1186/1752-153X-6-142

**Published:** 2012-11-23

**Authors:** Ang Gao, Xiaolan Yang, Chun Zhang, Gaobo Long, Jun Pu, Yonghua Yuan, Hongbo Liu, Yuanli Li, Fei Liao

**Affiliations:** 1 Unit for Analytical Probes and Protein Biotechnology, Key Laboratory of Medical Laboratory Diagnostics of the Education Ministry of China, College of Laboratory Medicine, Chongqing Medical University, Chongqing 400016, China

**Keywords:** *N*-hydroxysuccinimide esters, Monomethyl ether of poly-(ethylene glycol), Ethanolamine, 2,4,6-trinitrobenzenesulfonic acid, Molar equivalent

## Abstract

**Background:**

A new method is developed to quantify molar equivalents of *N*-hydroxysuccinimide (NHS) esters of derivatives of monomethoxyl poly-(ethylene glycol) (mPEG) in their preparations with NHS acetate ester as the reference.

**Results:**

NHS ester of succinic monoester or carbonate of mPEG of 5,000 Da was synthesized and reacted with excessive ethanolamine in dimethylformamide at 25°C for 15 min. Residual ethanolamine was subsequently quantified by absorbance at 420 nm after reaction with 2,4,6-trinitrobenzenesulfonic acid (TNBS) at pH 9.2 for 15 min at 55°C followed by cooling with tap water. Reaction products of ethanolamine and NHS esters of mPEG caused no interference with TNBS assay of residual ethanolamine. Reaction between ethanolamine and NHS acetate ester follows 1:1 stoichiometry. By the new method, molar equivalents of NHS esters of carbonate and succinic monoester of mPEG in their preparations were about 90% and 60% of their theoretical values, respectively. During storage at 37°C in humid air, the new method detected spontaneous hydrolyses of the two NHS esters of mPEG more sensitively than the classical spectrophotometric method based on absorbance at 260 nm of NHS released by reaction with ammonia in aqueous solution.

**Conclusion:**

The new method is favorable to quantify molar equivalents of NHS esters of mPEG derivatives and thus control quality of their preparations.

## Background

Monomethyl ether of poly-(ethylene glycol) (mPEG) is a pivotal biomaterial for formulating therapeutic proteins and similar biomolecules [1-6]. The modification of biomolecules with mPEGs is denoted PEGylation, and mPEGs are usually activated for selective PEGylation of amino groups of biomolecules under mild conditions [1,3,6,7]. *N*-hydroxysuccinimide (NHS) esters of succinic monoester of mPEGs (NHS-SC-mPEG), and of carbonate of mPEGs (NHS-CB-mPEG), are classical active forms for PEGylating accessible amino groups of biomolecules. For PEGylation, molar ratios of NHS esters of mPEGs to amino groups of biomolecules are primary determinants [7]. NHS esters of mPEGs are either synthesized in laboratories or commercial products; NHS esters of mPEGs and mPEGs are large polymers difficult to purify. More importantly, NHS esters of mPEGs easily undergo spontaneous hydrolyses during storage to yield mPEGs. The use of preparations of NHS esters of mPEGs with low purity for PEGylation of biomolecules brings more unwanted substances in PEGylated products to challenge the subsequent purification process. Hence, molar equivalents of NHS esters of mPEGs rather than mPEG lengths in commercial or laboratory preparations are their critical characteristics [8], and facile methods are needed for accurate analysis of molar equivalents of NHS esters of mPEGs in such preparations.

Chromatographic methods can validate NHS esters of mPEGs and quantify their molar equivalents [9,10], but are laborious and require a reference compound for each NHS ester of mPEG that is usually unavailable. Ionized NHS has an absorbance peak at 260 nm; molar equivalents of NHS esters of mPEGs can be quantified based on absorbance of NHS released by reactions of NHS esters of mPEGs with ammonia and/or hydroxide ion in alkaline aqueous solutions [9,11]. However, NHS esters of mPEGs undergo so fast reactions with ammonia and/or hydroxide ion that pre-existed NHS from spontaneous hydrolysis can not be accurately measured. Additionally, NHS is unstable in alkaline aqueous solutions and should be quantified within 20 min since reaction initiation. The glycyl-glycine test is commonly used to quantify molar equivalents of NHS esters of mPEGs [1,8,12,13]. However, it employs borate buffer at pH 8.0 for the reaction between glycylglycine and NHS esters; some NHS esters like that of mPEG-O-CH_2_-COOH undergo rapid hydrolyses at pH 8.0 and this glycyl-glycine test can not reliably quantify their molar equivalents. Hence, for controlling quality of preparations of NHS esters of mPEGs, new facile methods are still needed for accurate analysis of molar equivalents of NHS esters of mPEGs in such preparations.

In organic solvents free of water, NHS esters react rapidly and irreversibly with alkyl primary amines to yield amide derivatives and NHS, but basically undergo no spontaneous decomposition [10,14-17]. When an alkyl primary amine in an organic solvent can be facilely and reliably quantified in the presence of NHS and amide derivatives of mPEGs, the reactions between NHS esters of mPEGs and the alkyl primary amine in the organic solvent can be employed to quantify molar equivalents of NHS esters in preparations based on established stoichiometry of the reactions. The conjugate of 2,4,6-trinitrobenzenesulfonic acid (TNBS) with an alkyl primary amine has a strong absorbance peak around 420 nm and can be utilized for facile assay of the alkyl primary amine [18-20]. Herein, we reported a new facile method for quantifying molar equivalents of NHS esters of mPEG of 5000 Dalton (mPEG5k) in their preparations via reactions with ethanolamine and subsequent spectrophotometric assay of residual ethanolamine with TNBS.

## Results and discussion

### Selection of a suitable alkyl primary amine and a proper organic solvent

The designed method to quantify molar equivalents of NHS esters of derivatives of mPEGs in preparations requires a suitable alkyl primary amine, a proper organic solvent, an optimized concentration of the alkyl primary amine in the organic solvent and stoichiometry for the reaction between the primary amine and NHS esters of mPEGs. In appearance, any alkyl primary amine, any organic solvent compatible with NHS esters of mPEGs may be applicable. However, the consecutive reaction with TNBS to quantify the residual alkyl primary amine requires alkaline aqueous solutions, in which most alkyl primary amines and some organic solvents are incompatible. This complicated situation requires delicate selection of an organic solvent and an alkyl primary amine. Glycylglycine surely has unfavorable solubility in common organic solvents. Methylamine, ethylamine and propylamine are easily evaporated under room temperature. NHS esters have negligible reactivity with alcohol [15]. Hence, ethanolamine, glycine, ethylenediamine, *n*-butylamine with reasonable solubility in water were tested as candidate amines, while tetrahydrofurane (THF) and dimethylformamide (DMF) of unlimited solubility in water were compared as candidate organic solvents.

**Figure 1:**
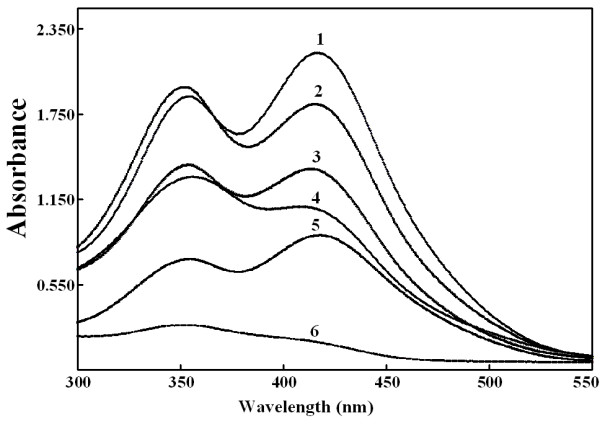
**Absorption spectra of conjugates of TNBS and some primary amines. **Each tested primary amine in the borate buffer for reaction with TNBS was 100 μmol/L, unless otherwise stated. The reaction continued at room temperature for 120 min. 1: Ethanolamine, 2: Glycine, 3: *n*-butylamine, 4: Ethylenediamine, 5: 100 μmol/L TNBS + 300 μmol/L ethylenediamine, 6: TNBS alone.

**Figure 2:**
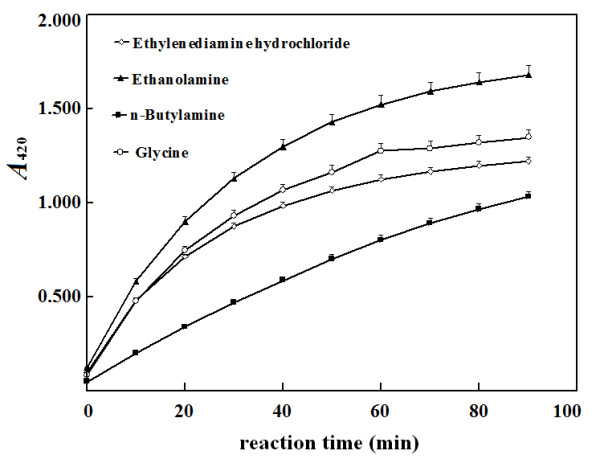
**Reaction process of TNBS with tested primary amines at room temperature.** Final concentration of each primary amine was 100 μmol/L for reaction with TNBS. Absorbance at 420 nm was measured at indicated periods with a proper reagent blank.

Those alkyl primary amines produced similar absorbance spectra after reaction with TNBS at 25°C for 120 min in alkaline aqueous solutions (Figure 1). To quantify an alkyl primary amine with TNBS, the absorbance at 420 nm was measured, but the conjugates of tested amines and TNBS had two absorption peaks, one around 420 nm while another around 350 nm. The conjugates of ethanolamine and *n*-butylamine with TNBS all had absorbance at 420 nm no less than that around 350 nm while that of glycine had slightly stronger absorbance at 350 nm. The situation with ethylenediamine is complicated. The conjugate of excessive ethylenediamine with TNBS had stronger absorbance at 420 nm than that at 350 nm, but ethylenediamine can not be quantified in this case. With TNBS in molar excess, both amino groups on ethylenediamine were conjugated with TNBS but the conjugate had stronger absorbance around 350 nm than that at 420 nm. These results made ethylenediamine unfavorable to quantify molar equivalents of NHS esters of mPEGs by the new method. On the other hand, in alkaline aqueous solution at 37°C, ethanolamine, glycine, ethylenediamine and *n*-butylamine displayed reaction rates with TNBS in a descent order (Figure 2). Solubility in water of ethanolamine or ethylenediamine ranks the first, that of glycine acts as the second while that of *n*-butylamine is the lowest. Solubility in THF and DMF of ethanolamine, ethylenediamine and *n*-butylamine is comparable, but that of glycine is too low for this new method to quantify molar equivalents of NHS esters of mPEGs. Hence, ethanolamine may be a suitable alkyl primary amine for the new method.

Both THF and DMF have no reactivity to NHS esters of mPEGs, and they are also good solvents for ethanolamine. When NHS carbonate ester of mPEG5k (NHS-CB-mPEG5k), or NHS ester of succinic monoester of mPEG5k (NHS-SC-mPEG5k), was used at a concentration over 1.0 mmol/L in THF plus 2.0 mmol/L ethanolamine, there were white sticky precipitates after reactions (Additional file 1: Figure S1, Supporting Material). But after the reaction mixture was diluted with water, such white precipitates disappeared. The mixture of ethanolamine and NHS at concentrations over 1.0 mmol/L in THF also produced such cloudy precipitates, but the mixture of either ethanolamine or NHS alone at the same concentration in THF produced no precipitates. However, the mixtures of NHS and ethanolamine at concentrations smaller than 60 mmol/L in DMF produced no such sticky precipitates. Hence, DMF is a suitable organic solvent for the new method. Ionic complexes might be formed between NHS (*p*K_a, aq_ = 6.1) and ethanolamine (*p*K_a, aq_ = 9.5) and precipitate in THF.

The reaction mixture of commercial DMF with TNBS produced negligible absorbance at 420 nm. The conjugate of TNBS with any of the tested primary amines has millimolar absorptivity over 10 (mmol/L·cm)^-1^ (Figure 2) [18-20]. Assuming the measurable absorbance below 1.500, the highest concentration of a suitable alkyl primary amine in DMF should be below 1.7 mmol/L if the reaction mixture is diluted by 21-fold for reaction with TNBS in an alkaline aqueous solution (the addition of just 50 μL DMF solution of ethanolamine and an NHS ester of mPEG as the sample to a total of 0.95 mL borate buffer plus 50 μL 0.4% aqueous solution of TNBS to quantify residual ethanolamine). For measuring absorbance below 1.200 at 420 nm with common spectrophotometers, final 0.83 mmol/L ethanolamine is employed in DMF to react with NHS esters of mPEGs for the new method.

**Figure 3:**
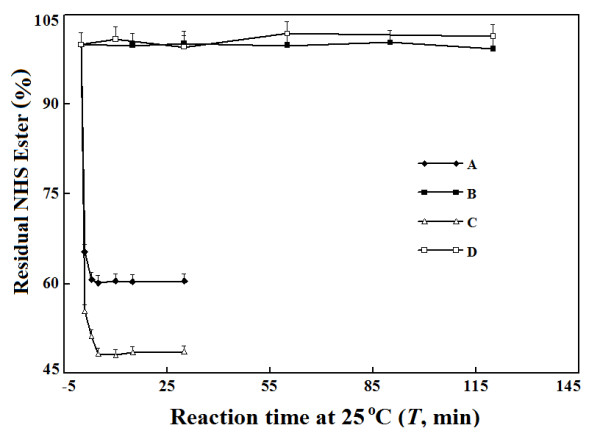
**Comparison of reaction rates of NHS esters of mPEG with amino and alcohol group. **To test reaction with amino group at 25°C, final ethanolamine concentration in DMF was 0.83 mmol/L. After the reaction for an indicated period, residual ethanolamine was quantified by withdrawing 50 μL sample for reaction with TNBS in borate buffer of 1.0 mL at 55°C for 15 min. The reaction mixture was cooled with tap water before assay of absorbance. The percentages of residual NHS esters are calculated and plotted versus reaction time. To test reaction with alcohol group at 25°C, final ethanol was 0.83 mmol/L in a total volume of 0.95 mL DMF containing an indicated NHS ester; after reaction for an indicated period, 50 μL diluted ethanolamine solution in DMF was added for final 0.83 mmol/L and 15-min reaction at 25°C. The residual ethanolamine was determined as described above. The percentages of residual NHS esters are calculated and plotted versus reaction time. **A**: NHS-SC-mPEG5k with ethanolamine; **B**: NHS-SC-mPEG5k with ethanol; **C**: NHS-CB-mPEG5k with ethanolamine; **D**: NHS-CB-mPEG5k with ethanol.

To quantify molar equivalents of NHS esters of mPEGs by this new method, there should be ethanolamine in molar excess to NHS esters of mPEGs. However, the use of ethanolamine to react with NHS esters of mPEGs faces the potential production of esters to complicate stoichiometry between NHS esters of mPEGs and amino group. When the differences in reaction rates of NHS esters of mPEGs with amino and alcohol groups in ethanolamine is large enough, the reactions of NHS esters of mPEGs with amino group in excess should be completed before significant formation of esters with alcohol group and thus the potential interference should be negligible. Indeed, within 15 min under room temperature, the reaction between ethanolamine and either of two NHS esters of mPEG5K was completed while there was negligible reaction between either NHS ester with ethanol at the same concentration in DMF (Figure 3). The reaction rate of NHS-AC with amino group in ethanolamine was even faster than those of NHS-CB-mPEG and NHS-SC-mPEG (Data not given). Therefore, the reactions of ethanolamine with NHS esters of mPEGs may be completed in 15 min under room temperature and still follow 1:1 stoichiometry to simplify the quantification of molar equivalents of NHS esters of mPEGs in preparations.

### Optimization of reaction conditions of ethanolamine with TNBS

**Figure 4:**
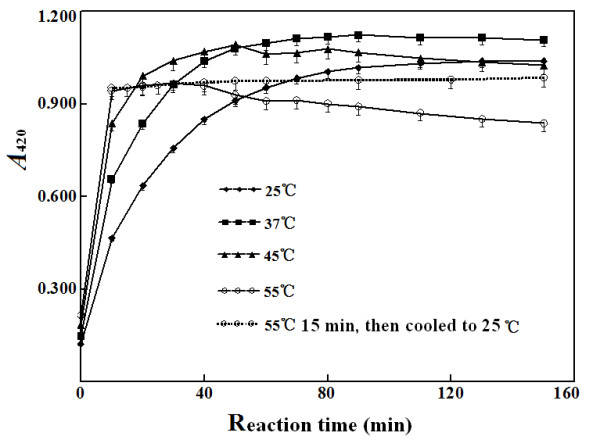
**Effects of reaction temperatures on reaction rates of TNBS with ethanolamine.** Except the differences in reaction temperatures, other conditions were the same. Final level of ethanolamine to react with TNBS was 100 μmol/L. At indicated time, reaction mixtures were withdrawn to measure absorbance at 420 nm.

The reaction of TNBS in excess with each of the tested alkyl primary amines at 25°C was not completed even after 90 min (Figure 2). The increase in reaction pH produced little improvement except increases in background absorbance. Ethanolamine has a high boiling point and its reaction with TNBS may be speeded up at higher temperatures. Indeed, the formation of the conjugates of TNBS and ethanolamine was greatly accelerated at 55°C so that the maximum absorbance was achieved after only 10-min reaction. Strangely, the absorbance at 420 nm of the conjugate of TNBS and ethanolamine began to decrease after 40-min reaction at 55°C or 90-min reaction at 45°C, but continued to increase for more than 120 min at 25°C (Figure 4). These decreases in absorbance at 420 nm may be owing to decomposition of conjugates at higher reaction temperature. As a result, after ethanolamine was reacted with TNBS at 55°C for 15 min, the reaction mixture was cooled to room temperature with tap water before the assay of absorbance at 420 nm. Since cooling, the absorbance at 420 nm remained constant within at least 120 min so that absorbance at 420 nm can be easily measured. This property makes the new method more advantageous over the classical spectrophotometric method based on the absorbance of NHS at 260 nm after reaction with ammonia or hydroxide ion in aqueous solutions [9,11].

**Figure 5:**
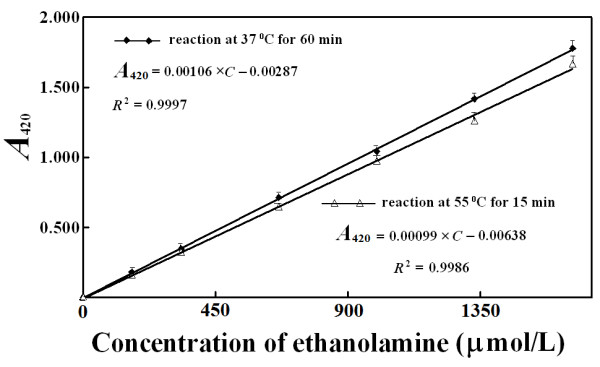
**Response of absorbance at 420 nm to ethanolamine quantities in reaction mixtures. A**: Reactions continued for 15 min at 55°C followed by cooling with tap water. **B**: Reaction was allowed to continue for 90 min at 37°C.

Potential interference from reaction products of ethanolamine and NHS esters of mPEG with the assay of ethanolamine was examined. In THF, NHS-CB-mPEG5k at 0.80 mmol/L and ethanolamine at 0.85 mmmol/L were mixed for 20-min reaction under room temperature. The amide was repetitively precipitated with 10-fold volume of diethyl ether and washed with THF till no ethanolamine in ether solution was detectable by reaction with TNBS. The amide of mPEG5k alone in DMF up to 1.0 mmol/L caused negligible absorbance at 420 nm after reaction with TNBS for 15 min at 55°C; they also caused no changes of absorbance at 420 nm for the conjugate of TNBS with ethanolamine. Similar results were observed with the amide from NHS-SC-mPEG5k. Moreover, NHS at concentrations smaller than 0.80 mmol/L in DMF did not alter the absorbance at 420 nm of the conjugate between TNBS and 0.83 mmol/L ethanolamine; it also caused no interference with the assay of other alkyl primary amines by reactions with TNBS (Additional file 1: Figure S2, Supporting Material). Therefore, the new method is resistant to pre-existed NHS and amide derivatives of mPEGs. Based on the reaction between ethanolamine and TNBS at 55°C for 15 min, the absorbance at 420 nm linearly responded to ethanolamine quantities with a slope about 92% of that after reaction at 37°C for 90 min (Figure 5). The difference in the response slopes may be attributed to increased spontaneous hydrolysis of TNBS and higher background absorbance at 55°C, but it resulted in no problems for the quantification of ethanolamine with TNBS.

Taken together, the optimized conditions for the new method are preset as follows. The reaction between 0.83 mmol/L ethanolamine in excess and a tested NHS ester of mPEG in DMF is allowed to continue for 15 min at 25°C for complete consumption of the NHS ester (Figure 3). The reaction between residual ethanolamine and TNBS in borate buffer at pH 9.2 continues for 15 min at 55°C followed by cooling with tap water to measure absorbance at 420 nm. These optimized conditions were used throughout, unless otherwise stated.

### Preliminary applications of the new method

**Figure 6:**
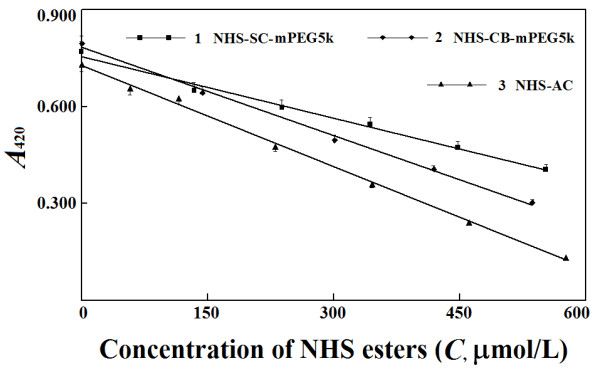
**Response plots of absorbance at 420 nm to quantities of NHS esters.** Reaction between NHS esters and ethanolamine continued for 15 min at 25°C; reaction between ethanolamine and TNBS continued for 15 min at 55°C; absorbance was measured after cooling of reaction mixture with tap water. The response equations were as follows. (1) NHS − SC − mPEG5k : *A*_420_ = − 0.0006 × C + 0.7548, *R*^2^ = 0.9911; (2) NHS − CB − mPEG5k : *A*_420_ = − 0.0009 × C + 0.7834, *R*^2^ = 0.9970; (3) NHS − AC : *A*_420_ = − 0.0010 × C + 0.7272, *R*^2^ = 0.9978.

Reference substances of NHS esters of mPEGs for this new method are difficult to prepare, which is more pronounced with chromatographic methods. NHS acetate ester as synthesized has satisfactory purity and can serve as a reference compound for the new method. In addition to labor and instrumentation, the difference in availability of reference substances makes this new method advantageous over chromatographic methods. For the reaction of NHS-AC with ethanolamine, the absorbance at 420 nm of reaction mixtures with TNBS negatively correlated to NHS-AC quantities within a reasonable range in DMF (Figure 6). The absolute value of the slope for such a response displayed a difference just about 1% from that for the response of absorbance at 420 nm to ethanolamine quantities. Therefore, the reaction between ethanolamine and NHS-AC follows 1:1 stoichiometry.

By TLC analysis, no free NHS was detectable in the two NHS esters of mPEG5k. By the new method, there was a linear decrease in absorbance at 420 nm to quantities of either of the two NHS esters of mPEGs in DMF for reactions with ethanolamine. Based on the response slopes and 1:1 reaction stoichiometry, NHS-CB-mPEG5k and NHS-SC-mPEG5k in their preparations were about 90% and 60% of their theoretical values calculated from their average molecular weights, respectively. By the classical spectrophotometric method based on absorbance at 260 nm of NHS released upon the reaction with ammonia, consistent molar equivalents of these two NHS esters of mPEG5k in their preparations were obtained, correspondingly (data not given). NHS-CB-mPEG5k in its preparation had a molar equivalent close to its theoretical value, supporting its high purity and the reaction stoichiometry of 1:1 between NHS-CB-mPEG5k and ethanolamine. It is a putative that NHS-SC-mPEG has reactivity with ethanolamine comparable to NHS-CB-mPEG5k [21,22]. The reaction between NHS-SC-mPEG and ethanolamine should be completed under the optimized reaction conditions; the lower molar equivalent of NHS-SC-mPEG in its preparation thus indicated its lower purity. Hence, both the new method and the classical spectrophotometric method are effective to check homogeneity of NHS esters of mPEGs in the absence of pre-existed NHS.

**Figure 7:**
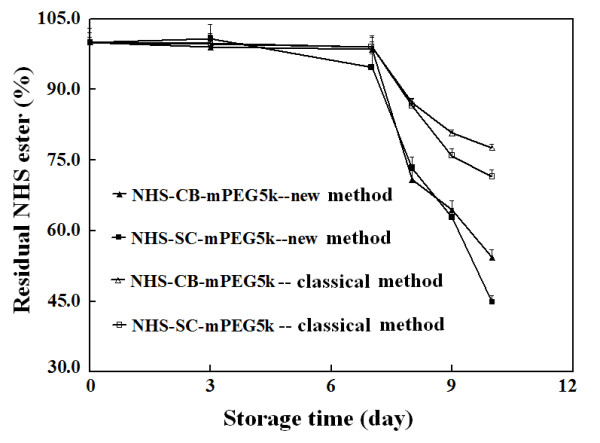
**Stability of NHS esters of mPEG5k under accelerated conditions. **Effects of two conditions were examined consecutively. In the first stage, samples in sealed bottles were kept at 37°C for one week. In the second stage, bottles were opened and samples were exposed to saturated humidity at 37°C in a closed cell culture incubator. At the same time in every indicated day for both stages, samples in bottles were dissolved with equal volumes of DMF to estimate molar equivalent of NHS esters under stated conditions.

To test the potential advantages of the new method to quantify molar equivalents of NHS esters of mPEGs in preparations containing pre-existed NHS due to partial hydrolysis, the storage stability of powder samples of NHS-SC-mPEG5k and NHS-CB-mPEG5k was examined under different conditions. As expected, both the new method and the classical spectrophotometric method gave consistent molar equivalents of NHS esters of mPEG5k in such powders when they were isolated from water during storage. After exposure of such powders to humid air for 6 h, the new method already can detect a statistical significant decrease in molar equivalents of two NHS esters of mPEG5k, but neither TLC analysis nor the classical spectrophotometric method based on NHS absorbance upon reaction with ammonia can safely detect hydrolysis of the two NHS esters of mPEG5k (data not given). After exposure of such powders to humid air for 24 h at 37°C, the two NHS esters display nearly 30% hydrolyses by the new method (Figure 7), less than 20% hydrolyses by the classical spectrophotometric method, and detectable hydrolyses by TLC analyses (Additional file 1: Figure S3, Supporting Material). After exposure of such powders to humid air for 72 h at 37°C, both NHS esters exhibited more than 50% hydrolyses by the new method, but less than 30% hydrolyses by the classical spectrophotometric method. The differences in percentages of hydrolysis in powder samples of each NHS ester of mPEG5k by these two spectrophotometric methods grew larger after exposure of these samples to humid air for more time. It is a putative that hydrolysis of NHS esters of mPEG5k produces NHS. Thus, for quantifying molar equivalents of NHS esters of mPEGs in preparation containing pre-existed NHS, the new method is superior to the classical spectrophotometric method.

In conclusion, a facile method is developed to quantify molar equivalents of NHS esters of mPEGs in laboratory or commercial preparations via their reactions with ethanolamine in dimethylformamide and subsequent spectrophotometric assay of residual ethanolamine with 2,4,6-trinitrobenzenesulfonic acid. The new method consumes just about 35 min for each analysis, displays resistance to pre-existed NHS, is universally applicable to common active esters as long as they are stable in DMF, and requires just one easily-accessible reference compound for different NHS esters. The glycylglycine test of active esters requires over 60 min for each analysis and is unreliable to active esters susceptible to hydrolysis [1,8,13]. The classical spectrophotometric method based on NHS absorbance at 260 nm is susceptible to the interference of pre-existed NHS originated from partial decomposition/hydrolysis of NHS esters. Taken together, the new method for controlling quality of commercial or laboratory preparations of active esters of mPEG derivatives and optimizing PEGylation process of therapeutic proteins is advantageous over other conventional methods.

## Materials and methods

### Chemicals and instruments

5% aqueous solution of TNBS and mPEG5k were from Sigma-Aldrich. NHS, triphosgene, glycine, *n*-butylamine, dicyclohexanylcarbodiimide (DCC), ethanolamine, ethylenediamine hydrochloride, and organic solvents were domestic reagents of analytical grade and used as received. Shimadzu UV 2550 and Mapada UV 1600 PC spectrophotometers were used.

### Syntheses of NHS active esters

**Scheme 1:**
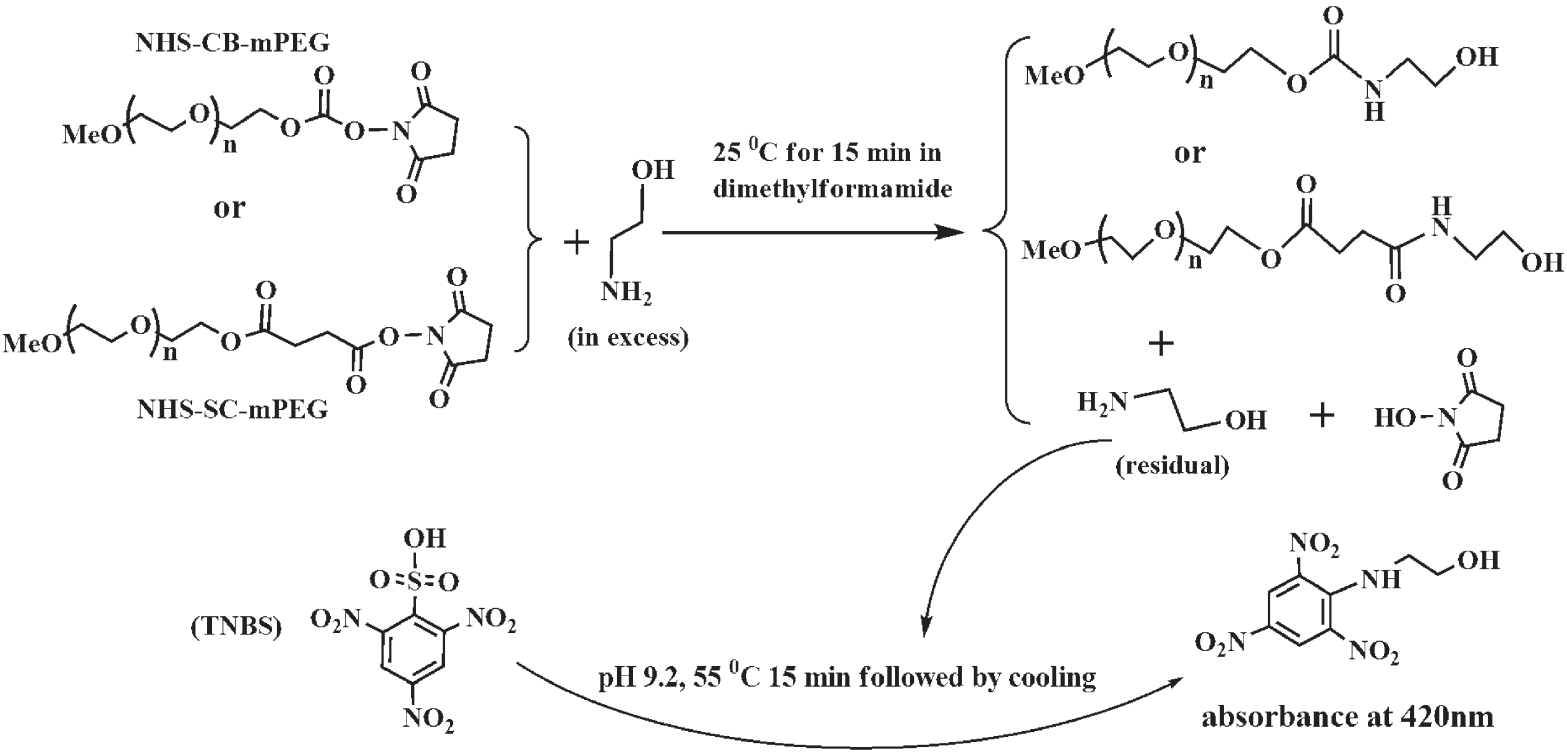
Synthesis route to NHS esters.

Three NHS esters were prepared as described below and frozen at below −10°C in sealed bottles prior to use, unless stated otherwise. (1) To 5.0 g mPEG5k in 10 mL dichloromethane, triphosgene (0.45 g) in 4 mL THF was added drop-by-drop and the mixture was stirred for 18 h at room temperature [21,22]. Then, the reaction mixture was concentrated to 5 mL under reduced pressure below 40°C, the intended intermediate was precipitated by 50 mL diethyl ether. The purification process, including the dissolution in 5 mL dichloromethane, the precipitation by 50 mL diethyl ether and wash with the ether, was repeated twice to give a white powder that was finally dissolved in 15 mL dichloromethane again. To this solution of the intermediate, 0.12 g NHS in 4 mL THF and 0.50 mL tri-(*n*-butyl)-amine were added consecutively for 8-h reaction at 37°C under continuous stirring. The reaction mixture was concentrated to about 5 mL under reduced pressure below 40°C; the resulting NHS carbonate ester of mPEG5k (NHS-CB-mPEG5k) was purified by repetitive precipitation by 50 mL diethyl ether followed by wash with diethyl ether for three times (Scheme 1). The overall yield of the final NHS esters was about 75% based on mass weight of mPEG5k used. (2) mPEG5k (5.0 g), succinic anhydride (0.12 g) and p-toluenesulfonic acid (50 mg) were melted at 70°C with oil bath for 4-h reaction. The resulting intermediate was dissolved in 10 mL THF, precipitated with 50 mL diethyl ether and washed repeatedly with ethyl ether; the purification process was repeated for three times. Then, to the intermediate in 15 mL THF, 0.12 g of NHS and 0.25 g of DCC were added for 8-h reaction under room temperature. After the removal of precipitates, the NHS ester of succinic monoester of mPEG5k (NHS-SC-mPEG5k) in THF was precipitated with 50 mL diethyl ether; the purification process was repeated for three times again [7]. The yield based on mass weight of mPEG5k used was about 75%. (3) Acetic acid (0.60 mL), NHS (1.2 g), DCC (2.4 g) were mixed in 15 mL THF for reaction under room temperature for 8 h. After the removal of precipitates, the solvent was removed under reduced pressure at room temperature; the residuals as white powder were washed repetitively with diethyl ether for more than five times. TLC analysis gave no detectable NHS in resulting NHS acetate, NHS-AC; the yield of NHS-AC was about 70% based on mass weight of NHS added.

### Reactions of NHS esters with primary amines and quantification with TNBS

Each NHS ester was reacted with an indicated alkyl primary amine in THF or DMF at room temperature for a stated period [11,14-17]. Then, 50 μL reaction mixture was withdrawn and mixed with 0.95 mL sodium borate buffer (200 mmol/L at pH 9.2); to this alkaline solution in 1.0 mL, a total of 50 μL TNBS aqueous solution (0.4%) was added and mixed by vortex. The mixture was kept at room temperature (25°C) or an indicated temperature for a stated period. Finally, absorbance of reaction mixture with TNBS at 420 nm (after cooling to room temperature) was measured using reagent blank to correct background absorbance [18-20]. NHS-AC was used as the reference owing to its high purity.

### Quantification of NHS esters based on NHS absorbance

The method completely followed those reported [9,11]. NHS ester was added to 0.10 mol/L aqueous ammonia and absorbance at 260 nm was measured from 5 min to 20 min since reaction initiation.

### Storage stability

An aliquot of THF solution of each NHS ester was transferred into a small bottle, dried in vacuum at 25°C and was then sealed. The effects of two conditions on storage stability were tested. Firstly, samples were kept at 37°C in sealed bottles. Secondly, samples were kept at 37°C in opened bottles in a closed cell culture incubator with saturated humidity. A bottle of powder sample was withdrawn at indicated time for analysis.

### Data analyses

Data were determined at least in triplicate and represented as mean ± standard deviation (SD). Coefficients of variation (CV) were below 10% unless otherwise stated. Student’s t-test was used for comparison.

## Abbreviations

DMF: Dimethylformamide; mPEG: Monomethoxyl poly-(ethylene glycol); NHS: N-hydroxysuccinimide; NHS-AC: NHS acetate ester; NHS-SC-mPEG: NHS esters of succinic monoester of mPEGs; NHS-CB-mPEG: NHS esters of carbonate of mPEGs; TNBS: 2,4,6-trinitrobenzenesulfonic acid; THF: tetrahydrofurane.

## Competing interest

The authors declared no competing interest.

## Authors’ contributions

AG, CZ, GL, JP, HL and YL performed the experiments; XY, FL, AG, CZ, YY, HL and YL analyzed experimental data; XY, AG, and FL wrote the manuscript; XY and FL conceived the idea. All authors read and approved the final manuscript.

## Supplementary Material

Additional file 1: Figure S1 Production of precipitates between NHS and ethanolamine. **S2.** Effects of NHS on TNBS assay of ethanolamine. **S3. **Spontaneous hydrolyses of NHS-SC-mPEG5k by TLC analyses. Click here for file
