# Supplementary material for: Facile spectrophotometric assay of molar equivalents of N-hydroxysuccinimide esters of monomethoxyl poly-(ethylene glycol) derivatives
Source: Chem Cent J. 2012 Nov 23;6:142. doi: 10.1186/1752-153X-6-142 (PMC3542108; doi:10.1186/1752-153X-6-142)
Supplement: Additional file 1: Figure S1 — Production of precipitates between NHS and ethanolamine. S2. Effects of NHS on TNBS assay of ethanolamine. S3. Spontaneous hydrolyses of NHS-SC-mPEG5k by TLC analyses. [file 1752-153X-6-142-S1.pdf]

Figure S1. Production of precipitates between NHS and ethanolamine

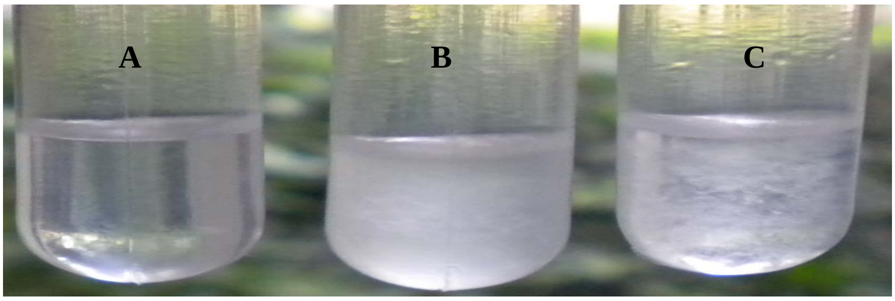

A: THF alone

B: NHS-CB-mPEG (1.5 mmol/L) with ethanolamine (1.5 mmol/L)

C: ethanolamine (1.5 mmol/L) + NHS (1.5 mmol/L)

Figure S2. Effects of NHS on TNBS assay of ethanolamine

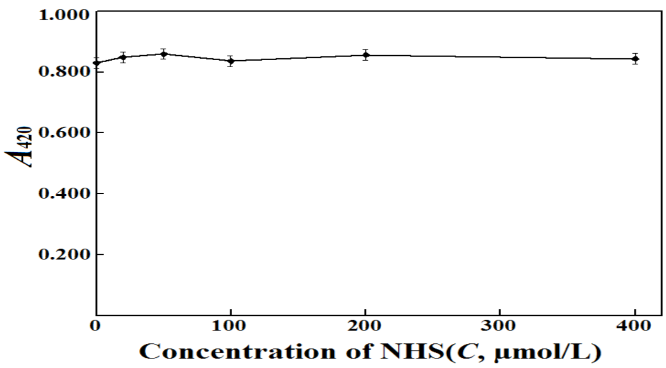

Figure S3. Spontaneous hydrolyses of NHS-SC-mPEG5k by TLC analyses

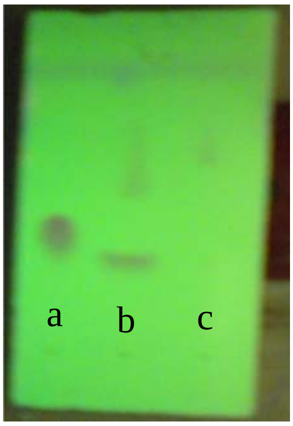

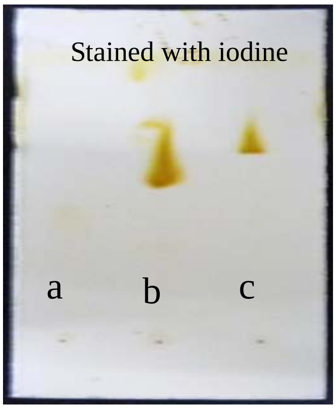

a: NHS alone, b: exposed to humid air for one day c: no exposure.
